# Innova 2020: A Follow-Up Study of the Fecal Microbiota of Infants Using a Novel Infant Formula between 6 Months and 12 Months of Age

**DOI:** 10.3390/ijms24087392

**Published:** 2023-04-17

**Authors:** Julio Plaza-Diaz, Francisco Javier Ruiz-Ojeda, Javier Morales, Rafael Martín-Masot, Eric Climent, Ángela Silva, Juan F. Martinez-Blanch, María Enrique, Marta Tortajada, Daniel Ramon, Beatriz Alvarez, Empar Chenoll, Ángel Gil

**Affiliations:** 1 Department of Biochemistry and Molecular Biology II, School of Pharmacy, University of Granada, 18071 Granada, Spain; 2 Instituto de Investigación Biosanitaria IBS.GRANADA, Complejo Hospitalario Universitario de Granada, 18014 Granada, Spain; 3 Children’s Hospital of Eastern Ontario Research Institute, Ottawa, ON K1H 8L1, Canada; 4 RG Adipocytes and Metabolism, Institute for Diabetes and Obesity, Helmholtz Diabetes Center at Helmholtz Center Munich, Neuherberg, 85764 Munich, Germany; 5 Institute of Nutrition and Food Technology “José Mataix”, Centre of Biomedical Research, University of Granada, Avda. del Conocimiento s/n. Armilla, 18016 Granada, Spain; 6 Product Development Department, Alter Farmacia SA, 28880 Madrid, Spain; 7 Pediatric Gastroenterology and Nutrition Unit, Hospital Regional Universitario de Málaga, 29010 Málaga, Spain; 8 ADM-BIOPOLIS, Scientific Park Universitat de València, 46980 València, Spain; 9 CIBEROBN (CIBER Physiopathology of Obesity and Nutrition), Instituto de Salud Carlos III, 28029 Madrid, Spain

**Keywords:** α-lactalbumin, arachidonic acid, *Bifidobacterium animalis* subsp. *lactis*, BPL1^TM^, docosahexaenoic acid, infant formula, body composition, postbiotics

## Abstract

The World Health Organization recommends exclusive breastfeeding on demand until at least the sixth month of life. Breast milk or infant formula is the infant’s primary food source until the age of one year, followed by the gradual introduction of other foods. During weaning, the intestinal microbiota evolves to a profile close to that of the adult, and its disruption can result in an increased incidence of acute infectious diseases. We aimed to determine whether a novel starting formula (INN) provides gut microbiota compositions more similar to those of breastfed (BF) infants from 6 to 12 months of age compared to a standard formula (STD). This study included 210 infants (70 per group) who completed the intervention until they reached the age of 12 months. In the intervention period, infants were divided into three groups. Group 1 received an INN formula with a lower protein content, a casein to whey protein ratio of approximately 70/30, twice as much docosahexaenoic acid as the STD formula, a thermally inactivated postbiotic (*Bifidobacterium animalis* subsp. *lactis*, BPL1^TM^ HT), and twice as much arachidonic acid as the STD formula contained. The second group received the STD formula, while the third group was exclusively BF for exploratory purposes. In the course of the study, visits were conducted at 6 months and 12 months of age. Compared to the BF and STD groups, the *Bacillota* phylum levels in the INN group were significantly reduced after 6 months. At the end of 6 months, the alpha diversity indices of the BF and INN groups differed significantly from those of the STD group. At 12 months, the *Verrucomicrobiota* phylum levels in the STD group were significantly lower than those in the BF and INN groups. Based on the comparison between 6 and 12 months, the *Bacteroidota* phylum levels in the BF group were significantly higher than those in the INN and STD groups. When comparing the INN group with the BF and STD groups, *Clostridium sensu stricto* 1 was significantly higher in the INN group. The STD group had higher levels of calprotectin than the INN and BF groups at 6 months. The immunoglobulin A levels in the STD group were significantly lower than those in the INN and BF groups after 6 months. Both formulas had significantly higher levels of propionic acid than the BF group at 6 months. At 6 months, the STD group showed a higher quantification of all metabolic pathways than the BF group. The INN formula group exhibited similar behavior to the BF group, except for the superpathway of phospholipid biosynthesis (*E. coli*). We hypothesize that the novel INN formula may promote an intestinal microbiota that is more similar to the microbiota of an infant who consumes only human milk before the weaning period.

## 1. Introduction

For infants, human milk is the preferred form of nutrition, and exclusive breastfeeding is strongly recommended for the first six months of life [1]. In both developing and developed nations, it is associated with excellent nutritional status, adequate growth and development, and a reduced rate of infant morbidity [2,3]. As a result, human milk has proven to be beneficial to the health of children as well as to the health of mothers [4,5]. It provides many nutrients, especially bioactive and immunogenic compounds that support immune system maturation and the protection of the intestines [6]. Although breastfeeding should last for 2 years, after infants are 6 months old, there is still a need for them to eat safe and suitable complementary foods [7].

The gut microbiota is assembled in a child’s first year of life during a maturation process. This is done by changing the positive and negative interactions between microbial taxa [8]. The aforementioned maturation is driven after the period of breastfeeding with the introduction of solid foods, which contributes gradually to a more suitable gut environment [9].

Despite the well-known benefits of breastfeeding, there are times, especially while weaning, when the mother might find it difficult or even impossible to breastfeed, which might result in the infant losing weight. In this context, infant formula can be used as a food source for adequate infant nutrition together with complementary foods. Within this context, the incorporation of novel food ingredients into infant formulas may contribute to their optimal growth and development [6,10]. The have been recommendations regarding infant formula composition published by organizations such as the American Academy of Pediatrics (AAP) and the European Society for Pediatric Gastroenterology Hepatology and Nutrition (ESPGHAN) [11]. There are also strict compositional and control standards for infant formula that have been established by the European Commission [12], the FAO/WHO Codex Alimentarius Commission [13], and the US Food and Drug Administration (FDA) [14]. Due to this, infant formulas are constantly being modified to provide added nutrients and bioactive compounds that have been identified in human milk [10].

Infancy weight gain and rapid growth are associated with obesity risk in adulthood [15]. The protein content of infant formulas is relatively high to ensure that all the essential amino acids are covered [16]. However, a high intake of protein early in infancy has been associated with obesity and increased metabolic risk later in childhood [15,17]. Therefore, the evaluation of infant formulas with a lower content of protein on growth and development compared to standard formulas is of great interest in pediatrics. Conversely, the relatively high levels of the long-chain polyunsaturated fatty acids (LC-PUFAs) of both the n-6 and n-3 series, which are generally found in human milk, have led to the incorporation of these nutrients into infant formulas in recent years. Arachidonic acid (ARA, 20:4 n-6) and docosahexaenoic acid (DHA, 22:6 n-3) are both present in human milk. DHA should, therefore, be included at 0.3–0.5% of the total fatty acids, along with a minimal amount of ARA equivalent to the amount of DHA [15,17].

Additionally, in recent years, research has been conducted on the use of prebiotics, probiotics, synbiotics, and postbiotics in infant formulas. Several studies demonstrated that the probiotic strain *Bifidobacterium animalis* subsp. *lactis* BPL1^TM^ can reduce fat accumulation in visceral adipose tissue in obese individuals [18,19,20] and its inactivated form (postbiotic BPL1^TM^ HT) modulates the gut microbiota composition [21]. Moreover, studies showed that increasing the use of probiotics in infants can help prevent several chronic diseases such as atopic eczema and necrotizing enterocolitis. As a result, infants’ health may improve in the short and long term [22,23]. It is currently necessary to evaluate the clinical outcomes of the addition of probiotics and postbiotics to infant formulas, and future studies should investigate their long-term effects.

Our previous article reported the results of a multicenter, randomized, blinded, controlled clinical trial examining the effects of a novel starting formula on weight gain and body composition in infants up to 6 and 12 months as well as its safety and tolerability compared to infants fed a standard formula (STD) and a reference breastfed (BF) infant group (INNOVA 2020 Study) [24]. The design and methodology were previously registered with Clinicaltrial.gov (NCT05303077) on 31 March 2022 and were updated and published on 7 April 2022 [25].

Compared to the STD (Nutriben^®^ Natal or STD), the novel formula (Nutribén^®^ Innova 1 or INN) contains less protein, a lower casein-to-whey protein ratio, and twice as much DHA/ARA. In addition, INN contains postbiotic BPL1^TM^ HT. Based on WHO guidelines, the INN formula is considered safe since weight gain and body composition were within normal limits. At 6 and 12 months, both formula groups gained more weight than a reference BF group, whereas no differences were found between the STD and INN groups. However, infants who received the INN formula experienced significantly fewer general disorders and disturbances than infants who received the STD formula. The STD formula group was significantly more likely to cause bronchiolitis, atopic dermatitis, and bronchiolitis [24].

In a previous report, we examined the impact of INN on the fecal microbiota composition of infants up to 6 months of age. In terms of the richness and diversity of the gut microbiota, the INN formula was more comparable to BF infants in terms of several genera, including *Clostridium*, *Lactobacillus Bacteroides*, and *Bifidobacterium*, as well as the calprotectin and short-chain fatty acid (SCFA) levels at 21 days, 2 months, and 6 months. Further, we found that the major metabolic pathways of bacteria were more similar between the INN formula and BF groups than between the STD formula and BF groups [26].

In the present work, we studied the fecal microbiota changes and associated metabolic pathways in the infants of the INNOVA 2020 Study between 6 and 12 months of life. Infants received either INN, STD, or exclusive BF together with complementary foods from 6 months onwards.

## 2. Results

### 2.1. Phyla and Genera Distribution

Table 1 depicts the differences according to the phylum in the interventions. It is pertinent to mention that the names of the phyla were updated recently. The updated taxonomic designations for *Actinobacteria*, *Bacteroidetes*, *Cyanobacteria*, *Firmicutes*, *Fusobacteria*, *Proteobacteria*, *Patescibacteria*, *Synergistetes*, and *Verrucomicrobia* are referred to as *Actinobacteriota*, *Bacteroidota*, *Cyanobacteriota*, *Bacillota*, *Fusobacteriota*, *Pseudomonadota*, *Candidatus Patescibacteria*, *Synergistetes*, and *Verrucomicrobiota*, respectively [27]. 

The *Bacillota* levels were significantly lower in the INN group than in the BF and STD groups after 6 months. The BF and INN groups had significantly different alpha diversity profiles than the STD group based on the alpha diversity indices measured at 6 months. Compared to both the BF and INN groups at 12 months, the *Verrucomicrobiota* levels were significantly lower in the STD group. When comparing 12 months to 6 months, significant differences were observed. The *Bacteroidota* levels were significantly higher in the BF group. In addition, there was a significant increase in the *Bacillota* levels in both the STD and INN groups. The *Pseudomonadota* levels were significantly decreased in all study groups. The study groups showed significant increases in all alpha diversity indices.

Table 2 shows the distribution of genera at 6 months and 12 months following the intervention. We found that *Bifidobacterium* was the main genus in all of the studied infants at 6 months of age. The STD group had significantly lower levels than both the BF and INN groups. There was a significant increase in *Clostridium sensu stricto* 1 in the INN group compared to both the BF and STD groups.

A significantly higher number of the *Ruminococcus gnavus* group, *Clostridioides*, *Flavonifractor*, and *Lachnoclostridium* genera were found in the STD group than in the BF or INN groups. The *Rothia* and *Escherichia-Shigella* genera were found at higher levels in the INN group than in either the BF or STD group. 

In comparison with the INN group, the BF and STD groups had significantly lower levels of *Veillonella* at 12 months. We observed a significant decline in the *Bifidobacterium*, *Veillonella*, *Enterococcus*, and *Escherichia-Shigella* genera from 6 to 12 months. In contrast, there was a significant increase in the *Blautia*, *Faecalibacterium*, *Lachnoclostridium*, *Anaerostipes*, *Ruminococcus*, *Subdoligranulum*, *Eubacterium hallii* group, and *Roseburia* genera. Both the BF and INN groups showed significant increases in *Collinsella* levels. The *Ruminococcus gnavus* group and *Flavonifractor* genera increased significantly in the INN group, while *Rothia* decreased significantly. It was found that *Clostridioides* decreased significantly in the STD group, while *Streptococcus* increased significantly. In addition, *Bacteroides* increased significantly in the BF group.

### 2.2. Rivera-Pinto Method Analysis

**Figure 1:**
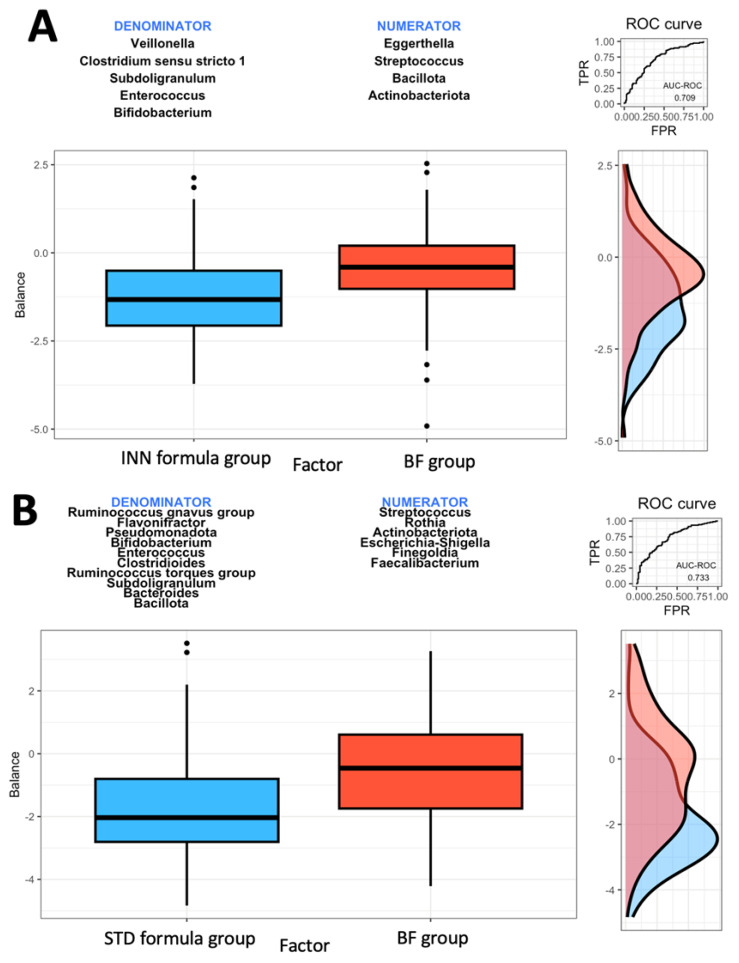

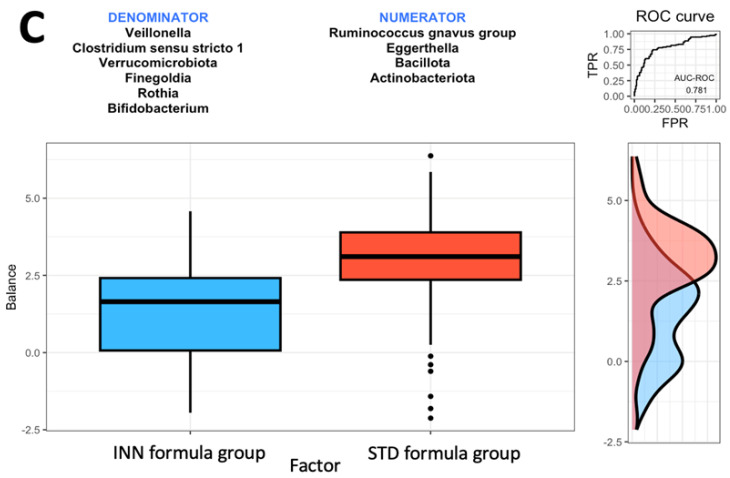
Group balances are presented in an overview. It is indicated at the top of the plot that two groups of taxa constitute the global balance. Box plots illustrate the distribution of balance scores for INN and BF groups (**A**), STD and BF groups (**B**), and INN and STD groups (**C**). On the right, the ROC curve with its AUC value and the density curve are displayed.

A microbial signature is generated using the geometric means of the data derived from two groups of taxa with relative abundances that are related to the response variable of interest, using the Rivera-Pinto method [28]. The *Bacillota* and *Actinobacteriota* phyla as well as the *Eggerthella* and *Streptococcus* genera were most closely associated with the BF group when compared to the INN formula group (Figure 1A). A higher balance score was associated with a significant relative abundance of the *Bacillota* and *Actinobacteriota* phyla and the *Eggerthella* and *Streptococcus* genera. This was when compared to the *Veillonella*, *Clostridium sensu stricto* 1, *Subdoligranulum*, *Enterococcus*, and *Bifidobacterium* genera. Figure 1A indicates a moderate discrimination accuracy between the INN formula group and the BF group based on the area under the curve (AUC) of 0.709.

Based on the comparison between the STD formula group and the BF group, it was found that the *Actinobacteriota* phylum and the *Streptococcus*, *Rothia*, *Escherichia-Shigella*, *Finegoldia*, and *Faecalibacterium* genera were most closely associated with the BF group (Figure 1B). The *Actinobacteriota* phylum and the *Streptococcus*, *Rothia*, *Escherichia-Shigella*, *Finegoldia*, and *Faecalibacterium* genera were associated with higher balance scores. This occurred when compared to the *Bacillota* and *Pseudomonadota* phyla and the *Ruminococcus gnavus* group, *Flavonifractor*, *Bifidobacterium*, *Enterococcus*, *Clostridioides*, *Ruminococcus torques* group, *Subdoligranulum*, and *Bacteroides* genera. Based on the AUC of 0.733, as shown in Figure 1B, the STD formula group and BF group exhibited a higher discrimination accuracy than that shown in Figure 1A (the AUC of 0.709). The last comparison was made between the two formulas. The *Bacillota* and *Actinobacteriota* phyla as well as the *Eggerthella* and *Ruminococcus gnavus* groups were associated with the STD formula group (Figure 1C). The AUC of 0.781 from Figure 1C indicates that the effects of both formulas on intestinal microbes differ. When compared to the *Verrucomicrobiota* phylum and the *Veillonella*, *Rothia*, *Clostridium sensu stricto* 1, *Finegoldia*, and *Bifidobacterium* genera (Figure 1C), the *Bacillota* and *Actinobacteriota* phyla and the *Eggerthella* and *Ruminococcus gnavus* groups had higher balance scores.

### 2.3. Calprotectin, IgA, and SCFAs

**Figure 2:**
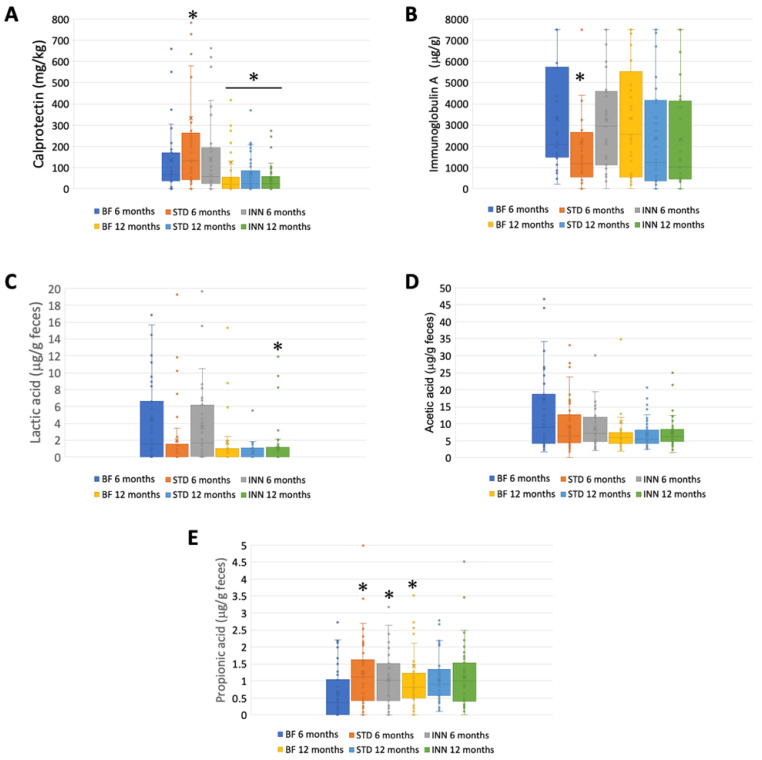
Calprotectin, IgA, and SCFAs. (**A**) Calprotectin (mg/kg). (**B**) Immunoglobulin A (μg/g). (**C**) Lactic acid (μg/g feces). (**D**) Acetic acid (μg/g feces). (**E**) Propionic acid (μg/g feces). * *p* < 0.05, significant differences were calculated with an independent sample median test adjusted by the Bonferroni correction for multiple tests.

Figure 2 illustrates the calprotectin, immunoglobulin A, SCFA, and lactate levels at 6 and 12 months for each group. The amount of calprotectin was significantly higher in the STD group than in the INN and BF groups at 6 months, decreasing significantly in all groups at 12 months (Figure 2A). Likewise, the STD group had significantly lower levels of immunoglobulin A than both the INN and BF groups at 6 months, with no evident differences at 12 months (Figure 2B). Regarding the SCFAs, no significant differences were found between the groups at 12 months. However, at 6 months, the lactic acid levels were lower in the STD group (although not significant), and, in both formulas, the propionic acid levels were significantly higher than those in the BF group. No differences in the acetic acid levels were detected among the groups at 6 months.

### 2.4. Metabolic Pathways

**Figure 3:**
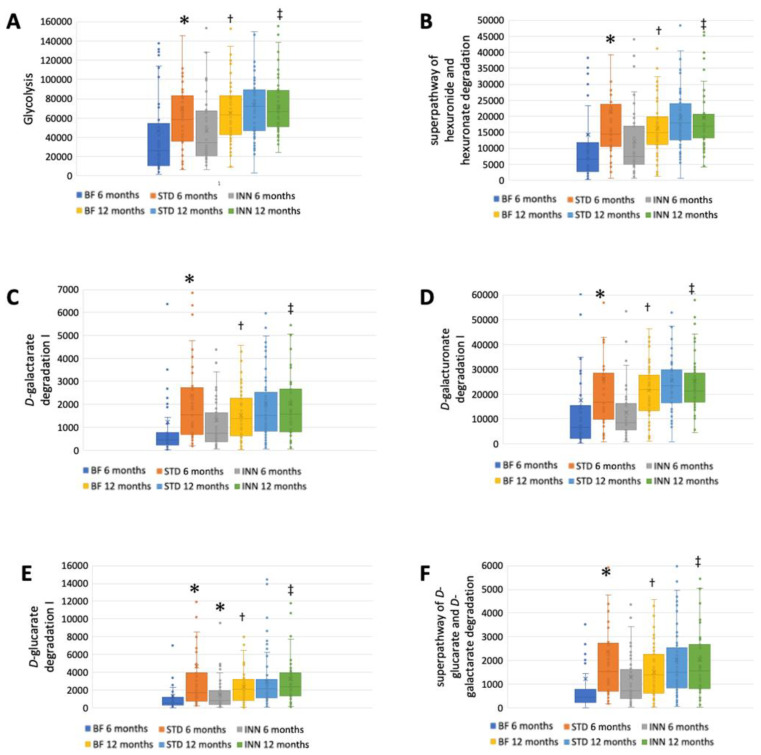

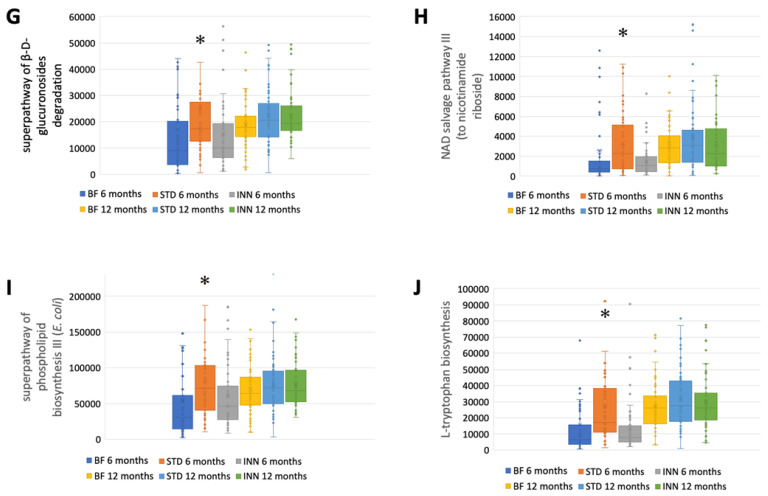
Major metabolic pathways. (**A**) Glycolysis. (**B**) Superpathway of hexuronide and hexuronate degradation. (**C**) D-galactarate degradation I. (**D**) D-galacturonate degradation I. (**E**) D-glucarate degradation I. (**F**) Superpathway of D-glucarate and D-galactarate degradation. (**G**) Superpathway of β-D-glucuronosides degradation. (**H**) NAD salvage pathway III (to nicotinamide riboside. (**I**) Superpathway of phospholipid biosynthesis (*E. coli*). (**J**) L-Tryptophan biosynthesis. * *p* < 0.05, significant differences were calculated with independent sample median test adjusted by the Bonferroni correction for multiple tests vs. BF group. ^†^ *p* < 0.05, BF group, 6 months vs. 12 months. ^‡^ *p* < 0.05, INN group, 6 months vs. 12 months.

The STD group showed several differences according to the metabolic pathways at 6 months compared to the BF group (Figure 3A–J). There were significantly higher abundances between the STD group and the BF group at 6 months in terms of glycolysis, the superpathway of hexuronide and hexuronate degradation, D-galactarate degradation I, D-galacturonate degradation I, D-glucarate degradation I, the superpathway of D-glucarate and D-galactarate degradation, the superpathway of β-D-glucuronosides degradation, NAD salvage pathway III (to nicotinamide riboside), the superpathway of phospholipid biosynthesis (*E. coli*), and L-Tryptophan biosynthesis. Compared to the BF group, only D-glucarate degradation was significantly higher in the INN group. At 12 months, no significant differences were observed.

A comparison between 12 months and 6 months showed that the BF group had an increased abundance of all metabolic pathways (Figure 3A–J). The INN formula group exhibited the same behavior as the BF group, with the exception of the superpathway of phospholipid biosynthesis (*E. coli*). Between 12 months and 6 months, no significant differences were observed in the STD group.

### 2.5. Correlations between Bacterial Diversity Indices, Bacterial Variables, SCFA Levels, Metabolic Traits, and Clinical Outcomes

According to Pearson’s correlations, there were some associations between the bacterial diversity indices, bacterial variables, SCFA levels, metabolic traits, and clinical outcomes in the BF, STD, and INN groups (Supplementary Figure S1).

After 12 months of intervention, the BF group showed several correlations between the microbiome and metabolic pathways. *Bifidobacterium* negatively correlated with the diversity indices (Shannon and Pielou’s evenness) and D-galacturonate degradation I, glycolysis, the superpathway of phospholipid biosynthesis (*E. coli*), and L-tryptophan biosynthesis. *Rothia*, *Eggerthella*, and *Veillonella* exhibited similar characteristics to *Bifidobacterium*, with negative associations with the major metabolic pathways. There was a negative association between *Actinobacteriota* and bronchiolitis (Supplementary Figure S1A).

At 12 months, *Bifidobacterium* maintained its negative correlation with the diversity indices (Shannon and Pielou’s evenness) and was only inversely correlated with L-tryptophan biosynthesis in the STD formula group. The biosynthesis of L-tryptophan was positively associated with *Clostridium sensu stricto* 1 and *Akkermansia*. The presence of *Collinsella* was negatively associated with cases of bronchiolitis (Supplementary Figure S1B).

At 12 months, *Bifidobacterium* was again negatively associated with the alpha indices (Shannon and Pielou’s evenness) and negatively associated with D-galacturonate degradation I and L-tryptophan biosynthesis. There was a positive correlation between *Collinsella* and glycolysis. L-tryptophan biosynthesis positively correlated with *Eggerthella* and *Akkermansia*. There was a negative association between *Veillonella* and glycolysis (Supplementary Figure S1C).

## 3. Discussion

The purpose of this study was to assess the differential effects on the intestinal microbiota of feeding infants with a novel INN compared to STD and breastfeeding at 6 months and 12 months of age, while the infants were weaning. A significant reduction in *Bacillota* levels was observed in the INN group after 6 months compared to the BF and STD groups. At 6 months, the alpha diversity indices measured in the BF and INN groups were significantly different from those in the STD groups. The *Verrucomicrobiota* levels in the STD group were significantly lower than those in the BF and INN groups at 12 months. The comparison between 6 and 12 months showed that in the BF group, the levels of *Bacteroidota* were significantly higher compared to the INN and STD groups. In both the STD and INN groups, the *Bacillota* levels increased significantly. In all study groups, the *Pseudomonadota* levels were significantly reduced. All the alpha diversity indices increased significantly in all study groups. In all of the infants studied at 6 months of age, *Bifidobacterium* was the dominant genus. STD group had significantly lower levels than the INN group. Comparing the INN group to the BF and STD groups, *Clostridium sensu stricto* 1 was significantly higher in the INN group. Following the Rivera-Pinto approach, the INN formula group was more similar to the BF group than the STD group. The superior AUC value was found in a comparison of the two formulas. At 6 months, the STD group had higher calprotectin levels than the INN and BF groups. In the STD group, the immunoglobulin A levels were significantly lower than those in the INN and BF groups after 6 months. At 6 months, the propionic acid levels were significantly higher in both formulas than in the BF group. After 12 months, there was no difference between the study groups. The STD group showed a higher quantification of all the evaluated metabolic pathways than the BF group at 6 months. Compared with the BF group, only D-glucarate degradation was significantly higher in the INN group. There were no significant differences observed at 12 months. Comparing the 6-month and 12-month results of the BF group, it was found that all the metabolic pathways increased in the BF group. Except for the superpathway of phospholipid biosynthesis (*E. coli*), the INN formula group exhibited similar behavior to the BF group. The STD group did not show any significant differences between 12 months and 6 months.

An adequate diet in infancy and early childhood is essential to the development, growth, and health of children [29]. As a result of this, diet (breastmilk, infant formula, and complementary foods, among others) plays an instrumental role in fulfilling an individual’s nutritional and physiological requirements, such as vitamins and minerals, which cannot be synthesized by humans [30]. It is critical to emphasize that breastfeeding should continue until the child reaches the age of two years. This is in addition to the introduction of complementary foods after 6 months [31,32]. 

In our study, the main comparison was made using the BF group. A detailed description of the effects of the different groups on weight gain, body composition, safety, and tolerability has been provided elsewhere [24].

It is critical to emphasize that breastfeeding should continue until the child reaches the age of two years. This is in addition to the introduction of complementary foods after 6 months [31,32].

In previous work, other authors reported that the gut microbiota of 24 Canadian term infants at 4 months of age was dominated by *Actinobacteriota* (mainly *Bifidobacterium*) and *Bacillota* (with diverse representation from numerous genera) [33,34]. The gut microbiota has a significant impact on long- and short-term health. *Bifidobacterium* plays a critical role in maintaining host homeostasis during early life, as one of the most abundant genera within the infant intestinal microbiota [35]. During the first 6 months after birth, *Bifidobacterium* is typically the dominant microbial taxon [36]. However, its relative abundance decreases after weaning [37], with the *Actinobacteriota* and *Bacillota* phyla representing more than 90% of the relative abundance afterward. In our study, the main genus present in all of the infants studied at 6 months of age was *Bifidobacterium*, which decreased significantly at 12 months in all groups. Even though another study evaluating the fecal microbiota of Australian newborns showed a significant increase in the *Bifidobacteraceae* family and several strains of *Bifidobacterium* after the consumption of infant formula [38], most publications point to a decrease in bifidobacteria after weaning [39,40,41,42,43,44].

Although different technologies have been used to compare the microbiota of breastfed and formula-fed infants [45,46,47], 16S rRNA remains the gold standard. Using fluorescent in situ hybridization in conjunction with flow cytometry, other authors found that the most predominant detected group was *Bifidobacterium*, followed by *Enterobacteriaceae* and *Bacteroides*, regardless of whether the infant was breastfed or formula-fed. Infants who were formula-fed were only associated with the *Lactobacillus* group [45].

At 6 months, the INN formula group had similar alpha diversity indices as the BF group. At 12 months, all groups showed a significant increase in alpha diversity. This is similar to other studies that compare the fecal microbiota of infants from 6 months to 12 months [48] and from 6 months to 24 months [49].

*Verrucomicrobiota* spp. and *Clostridiales* spp., members of the intestinal microbiota, are associated with increased expression levels of *il10* and *tgfb* as well as the Treg transcription factor FOXP3 in an animal model, and the potential induction of regulatory immunity in the host was speculated [50]. In addition, the *Verrucomicrobiota* levels were significantly lower in the STD group than in the BF and INN groups.

The Rivera-Pinto algorithm is used to identify microbial signatures [28]; based on an individual’s specific microbiome, these signatures can be used to diagnose, prognosticate, and predict the therapeutic response [28]. Based on the Rivera-Pinto approach [28], we observed that the INN formula group has a higher AUC value than the BF group, all of which are compared using the ROC curve. The safety of the INN formula group and its impact on the body composition group was described elsewhere [24,25]. It is believed that the modifications made to the INN formula are intended to produce a similar effect to breastfeeding. 

A growing body of research indicates that gut colonization after birth has a long-term “programming” effect on health. In light of this, it has been hypothesized that gut microbiota plays a significant role in modulating the immune system, and, as a consequence, it has a critical role in preventing infections in infants. Several factors influence this colonization process, including feeding habits [51]. In general, infants who have been breastfed tend to possess bifidobacteria as the predominant microorganisms and develop a slower level of intestinal microbiota structural diversity than infants fed a standard formula, as their microbiota is more diverse and contains higher proportions of bacteria such as *Escherichia coli*, *Clostridium*, and *Bacteroides* [46].

Calprotectin is a calcium-binding protein of the S-100 protein family that is predominantly found in neutrophils and throughout the human body. Calprotectin is present in feces as a result of neutrophil migration into the gastrointestinal tract as a result of inflammation. As a biomarker for gastrointestinal disorders, fecal calprotectin concentrations are well correlated with intestinal inflammation [52]. There was a higher level of calprotectin in the STD group after 6 months compared to the INN and BF groups. This result may be related to the reformulation of the INN formula and its similarity to the BF group. In the gastrointestinal, respiratory, and genitourinary tracts, immunoglobulin A functions as the dominant antibody of immunity for mucosal homeostasis [53]. In the body, this type of immunoglobulin is the second-most-abundant type and plays an instrumental role in protecting against antigens [54]. As a result of its many systemic functions, immunoglobulin A is produced at a much higher rate than all other immunoglobulin subtypes. After 6 months, immunoglobulin A levels were significantly lower in the STD group than in the INN and BF groups. Once again, the STD formula displayed significant differences from the BDF formula and the INN formula. Furthermore, all study groups showed significant decreases in calprotectin levels after 12 months, possibly as a result of the introduction of complementary foods.

During 6 to 12 months of intervention, the BF group demonstrated increased levels of the various metabolic pathways. Degradation, the formation of different metabolites, and lipid and protein synthesis are among the different processes. The process of glycolysis involves the conversion of a six-carbon glucose molecule into two three-carbon keto acids (pyruvate) [55]. D-glucarate is an effective antitumoral agent able to bind to environmental carcinogens such as benzo[a]pyrene [27,56,57]. The natural substance galactarate is a dicarboxylic acid analog of D-galactose. *E. coli* is capable of using both galactarate and D-glucarate as carbon sources for growth. By interacting with coenzyme A through the pyruvate dehydrogenase complex, pyruvic acid produces carbon dioxide, NADH, and acetyl CoA. In phosphoenolpyruvate synthetase, pyruvic acid interacts with water and ATP, resulting in the release of hydrogen ions, phosphates, AMPs, and phosphoenolpyruvic acids [58,59]. The superpathway of phospholipid biosynthesis III (*E. coli*) is involved in the synthesis of lipopolysaccharides. In humans and other animals, this elicits a strong immune response (and contributes to Gram-negative septic shock), which is detected at picomolar levels by the toll-like receptor-4 receptor found in the innate immune system [60]. A variety of anabolic pathways are influenced by the metabolism of tyrosine, phenylalanine, and tryptophan [61].

In comparison with the BF group, only the INN formula group showed a similar metabolic pattern. The metabolic pathways were all increased at 12 months, following the inclusion of complementary foods and the development of metabolic processes and the immune system. In contrast, the STD formula group showed upregulation of that process in the 6 months after the intervention, without statistical changes at the end.

### Limitations and Strengths

In this study, the primary strength is its design, which is a randomized, multicenter, double-blind, parallel, comparative clinical trial comparing two starting infant formulas for infants based on very strict eligibility, inclusion, and exclusion criteria. The hypotheses were that the weight gain among infants fed the INN formula would be similar to that observed among children fed the STD formula. For exploratory analysis, a third group of unblinded BF infants was also used.

The study does, however, have several limitations. First of all, the fecal microbiota diversity and bacterial groups were not initially calculated based on the number of infants per group. Rather, they were calculated in order to assess differences in growth rates. In order to obtain significant differences in certain underrepresented bacterial groups, the number of infants examined in the present study would need to be higher. There is another limitation associated with the microbiota methodology. It is important to note that, in our study, we used a methodology that involves the amplification of specific segments of the 16S ribosomal RNA of bacteria; however, sequencing the whole gene would allow for more accurate identification of the microbial species beyond families and genera. As well, the variation in the fecal microbiota cannot be attributed to a specific component of the experimental formula, since it differs from standard milk formulas in several respects. 

We recommend that new longitudinal studies be conducted between birth and weaning using sufficient numbers of infants fed formulas that differ from standard formulas by a single ingredient. Nutrients, probiotics, or postbiotics that have a significant impact on intestinal microbiota should be considered in these studies. Recent research indicates that dietary patterns are consistently correlated with bacterial groups that play a similar role in both health and disease [62]. Moreover, specific foods and nutrients are associated with species that confer mucosal protection and anti-inflammatory effects [62].

## 4. Materials and Methods

### 4.1. Ethical Considerations

Throughout this clinical trial, the ethical–legal principles established in the latest revision of the Declaration of Helsinki, the recommendations of the International Conference on Harmonization Tripartite, and the current regional regulations governing pharmacovigilance and food safety were followed. This study was approved by the Committee for Technical Investigation in Regional Medicine in the Madrid Community (CEIm-R) on 11 May 2018 under the name INNOVA 2020 version 2.0 (EC 42.18). During the course of this study, all personal information was kept confidential. The data were processed in accordance with the Spanish Organic Law 3/2018, of 5 December 2018, concerning the protection of personal data and the guarantee of digital rights. Researchers or institutions involved in the study provided direct access to the data or source documents for monitoring, auditing, and review by CEIm-R. The health authorities were also permitted to inspect the trial. Ruiz-Ojeda et al., 2022, and Plaza-Diaz et al., 2023, provide further details regarding the study protocol [24,25].

### 4.2. Design of the Trial

The objective of INNOVA 2020 was to evaluate the equivalence of two starting formulas for infants through a multicenter, randomized, double-blind, parallel, comparative study. A third unblinded group of BF infants was used as an additional reference group for exploratory analysis. There was a common label on both infant formulas, ensuring the blinding of the investigators and participants. The current EU legislation (EC Regulation No. 1924/2006) does not require specific clinical tests to demonstrate that infant formulas are nutritionally and healthfully beneficial [25].

In the study, a pediatrician informed parents that their children could participate as early as 15 days of age. As soon as they agreed to participate, they attended the health center for a baseline visit a week later (at 21 days of age). The pediatrician requested relevant information from the mother’s medical history during the 15-day meeting with the parents. It was necessary to obtain this information for the study, and it was not included in the pediatric history. When a visit took place within the scheduled time, it was considered valid with a margin of 3 days for a 21-day visit, 1 week for a 2-month visit, and 2 weeks for a 4-, 6-, or 12-month visit. More information regarding inclusion and exclusion criteria, withdrawal of participants, and study procedures can be found in Ruiz-Ojeda et al., 2022 [25].

According to the guidelines published by the AAP Task Force on Clinical Testing of Infant Formula [63], the study sample size is 210 children (70 per group) based on the main outcome of weight gain. Pediatricians recruited infants through active and consecutive recruitment methods. The study was conducted in 21 centers, all located in Spain, of which 17 centers recruited a minimum of one subject. A total of 217 subjects signed the informed consent form, and then 145 were randomly assigned to receive one of the two infant formulas. Among these 145, 3 failures were caused by randomization failures and 2 by screening failures. As a result, 140 infants who met all inclusion criteria and no exclusion criteria were included in the study, and 70 of those infants were unblinded among exclusively BF infants. In total, 185 subjects completed all study visits. Twenty-five dropouts occurred in the BF group, eight in the INN group, and give in the STD group. Clinicaltrials.gov registered the trial (NCT05303077, https://clinicaltrials.gov/ct2/show/NCT05303077, accessed on 1 April 2023) on 31 March 2022, which was last updated on 7 April 2022.

### 4.3. Characteristics of the Formula

o Group 1: Nutribén^®^ Innova 1 (INN; infant formula 1)
o Group 2: Nutribén^®^ Standard (STD; infant formula 2)
o Group 3: BF (Exploratory analysis with external controls)

Infants were recruited from primary care pediatric clinics by the pediatricians participating in the trial. A pediatrician informed and invited parents of 15-day-old infants who regularly visited their offices for routine medical examinations to participate in the study. In the formula-feeding group, infants who were unable to BF (for a variety of reasons) were invited to participate. For every two infants supplemented with infant formula, a candidate BF subject was recruited at each center. This was necessary in order to maintain balance between the three arms of the study. Infants who met the inclusion and exclusion criteria of the study were included in the BF group [25].

The experimental product object of this trial (INN) and the STD formula comply with the recommendations of the ESPGHAN and with Regulation 609/2013 of the European Parliament and of the Council regarding foods intended for children, infants, and young children; foods for special medical purposes; and complete diet substitutes for weight control [64]. The supplementary information (Supplementary Table S1) provides a detailed description of the composition of each of the products. Both formulas were given ad libitum to the infants. The two trial formulations were administered in accordance with the instructions of the manufacturer, as contained in the package insert.

### 4.4. Sampling Process

To ensure the stability of the fecal samples, ADM-Biopolis provided a kit that included a buffer for sample stabilization. Durviz S.L., Valencia, Spain, provided a collector tube with components of 0.02 M EDTA, 0.025 M sodium citrate dihydrate, and 5.3 M ammonium sulfate (product reference, RBMST02). In accordance with the specifications of the manufacturer, DNA remains stable at room temperature (15–25 °C) for several months and at −20 °C or −80 °C indefinitely. Following the original codes provided by the researchers, the samples were processed and sequenced, and the group was assigned in the final bioinformatics analysis.

### 4.5. Extraction of DNA

Following a modified protocol from Yuan et al. [65], an optimized DNA extraction protocol was applied using the QIAmp Power Fecal Kit (Qiagen, Hilden, Germany). To ensure that the DNA met the minimum extraction conditions, DNA quality control was conducted using Nanodrop equipment (ThermoFisher, Madrid, Spain). A260/230 nm for salt and phenol contamination and A260/280 nm for protein contamination were measured spectrophotometrically to determine DNA yield. 

### 4.6. Analysis of Sequences and Bioinformatics

A PCR amplification of the extracted DNA was performed using primers that target the hypervariable regions of 16S, V3, and V4 [66] and a primer for dimer cleanup. The libraries were sequenced using Illumina’s Novaseq 6000 platform and 250PE. A negative control containing water was obtained to confirm that there was no contamination. Furthermore, a second negative control consisting of DNA isolated from a collector tube without a sample was sequenced as a control, and the results were also negative.

Raw sequences were demultiplexed using Illumina bcl2fastq2 Conversion Software v2.20, and raw data were imported into QIIME 2 2020.8 open-source software [67] by using the q2-tools-import script that uses PairedEndFastqManifestPhred33 input format. DADA2 [68] was used to denoise sequence variances, each of which differed by one nucleotide, using a quality-aware model of Illumina amplicon errors. Following the retrieval of the quality scores, the q2-dada2-denoise script was run to truncate and trim the forward reads at position 288 and position 6. We trimmed the reverse reads at position 7 after truncating them at position 220. A consensus filter was used to remove chimeras, which detects chimeras in samples individually and removes those that appear in a sufficient number of samples. Additionally, forward and reverse reads were merged during this step. Amplicon sequence variants (ASVs) aligned with MAFFT [69] via q2-alignment were used to construct phylogenies using FASTTREE2 (via q2-phylogeny) [70]. A naive Bayes taxonomy classifier was applied (via q2-feature-classifier) [71] against SILVA 16S V3-V4 v132_99 [72]. Samples with fewer than 10,000 reads were excluded from the data filtering process. No samples were excluded from our study.

Using the vegan library [73], the diversity of the samples was examined. In addition to Shannon, Simpson, and species richness, Pielou’s evenness indices were examined as part of the study.

### 4.7. Functional Profiles

PICRUSt2 [74] was used to predict the potential functional profiles of the sequenced samples. A reference tree containing 20,000 16S rRNA genes from prokaryotic genomes in the Integrated Microbial Genomes (IMG) database was constructed from phylotypes. Using the Clusters of Orthologous Groups of Proteins (COG) and Enzyme Commission numbers (EC) databases, functional annotations were performed on these genomes. To determine MetaCyc pathways, EC numbers were regrouped into MetaCyc reactions. In order to calculate pathway abundances, the harmonic mean of the key reaction abundances in each sample was used. To calculate the abundance of each gene family per sample, we corrected the abundance of phylotypes by the copy number of the 16S rRNA gene. As a result, we multiplied the copy number by the predicted function.

### 4.8. Biochemical Analysis

An ELISA kit was used to determine the levels of Calprotectin and IgA, following the instructions of the manufacturer (Immundiagnostik AG, Bensheim, Germany). Lactic acid and SCFAs (acetic, butyric, and propionic acids) were determined using high-performance liquid chromatography (HPLC). An Alliance 2695 HPLC system coupled to a refractive index detector (RID, 2414, Waters Corp., Milford, MA, USA) was used. The column used was an Aminex HPX-87H (300 mm × 7.8 mm) from Bio-Rad (Hercules, CA, USA) at a temperature of 60 °C. Isocratic elution was performed with 5 mM H_2_SO_4_ at a flow rate of 0.6 mL min^−1^. Identification was performed by comparison with the retention time of standards, and calibration curves were used for quantification.

### 4.9. Rivera-Pinto Analysis

A Rivera-Pinto analysis identifies microbial signatures, i.e., groups of microbes that predict phenotypes of interest. These microbial signatures may be used to diagnose, prognosticate, or predict therapeutic responses based on an individual’s unique microbiota. As a consequence, identification of microbial signatures requires both modeling and variable selection, i.e., modeling the response variable and selecting the taxa that provide the highest level of classification or prediction accuracy. We evaluated specific signatures at the phylum and genus levels as part of the Rivera-Pinto method and Selbal algorithm, a method of selecting a sparse model that adequately explains the response variable of interest. Based on data collected from two groups of taxa, geometric means are used to calculate microbial signatures. As the name implies, these groups are those with relative abundances, or balances, that are related to the response variable of interest [28].

### 4.10. Statistical Analysis

Data are expressed as medians and ranges. Using the independent sample median test, the *p*-value was calculated. The different letters indicate significant differences (*p* < 0.05) calculated using an independent sample median test and Bonferroni correction for multiple testing.

The functional pathways profile data and the SCFAs are presented as means and standard errors of the means. According to the independent sample median test, *p* < 0.05 was considered statistically significant. Rivera-Pinto and Selbal algorithms were applied to identify specific signatures at phylum and genus levels; based on geometric means of data from two groups of taxa with relative abundances, or balances, that are related to the response variable of interest, this method identifies microbial signatures [28].

This study examined the relationships between diversity indices, microbiome variables, metabolic parameters, SCFA levels, and clinical outcomes using Pearson’s correlations (bronchiolitis and gastrointestinal symptoms were published elsewhere [24], as these symptoms were assessed after 6 months of the intervention). R Studio’s corrplot function [75] was used to express associations by correcting multiple testing with the FDR procedure [76]. The graph shows only significant and corrected associations. In the graphs, red and blue lines indicate the correlation values, with negative correlations highlighted in red (−1) and positive correlations highlighted in blue (+1) (Supplementary Figure S1).

## 5. Conclusions

As a result of our study, we found that the INN formula had a similar impact as the BF formula on the fecal microbiota, in silico metabolic pathways, and certain biochemical parameters related to immunity at 12 months. The addition of novel ingredients to starting formulas represents a growing field of research that should always be evaluated using randomized clinical trials. Future intervention studies should investigate the mechanisms of microbial action through which the diet affects the development of the gut during the first year of life and beyond.

## Figures and Tables

**Table 1:** Distribution of phyla and alpha diversity indices.

Phylum	6 Months			12 Months			*p*-Value		
	BF (n = 52)	STD (n = 55)	INN (n = 52)	BF (n = 59)	STD (n = 63)	INN (n = 60)	BF	STD	INN
*Actinobacteriota*	88.4 (4.3–99.1)	80.5 (1.5–98.6)	92.5 (6.0–98.0)	69.2 (8.5–97.6)	65.2 (6.6–96.6)	64.0 (1.9–95.9)	0.155	0.065	0.155
*Bacillota*	10.2 (0.8–95.6) ^a^	15.8 (1.2–98.3) ^a^	6.9 (1.3–93.8) ^b^	28.5 (1.3–90.9)	33.5 (2.2–93.1)	34.6 (3.0–97.2)	0.105	0.003	<0.001
*Verrucomicrobiota*	0.05 (0–10.7)	0.05 (0–66.6)	0.04 (0–35.3)	0.04 (0–15.2) ^ab^	0.03 (0–9.6) ^a^	0.07 (0–29.7) ^b^	0.509	0.078	0.509
*Pseudomonadota*	0.2 (0–1.1)	0.2 (0.003–0.8)	0.3 (0.01–1.2)	0.1 (0–1.0)	0.1 (0–1.0)	0.1 (0–1.3)	0.018	0.044	0.018
*Bacteroidota*	0.02 (0–1.0)	0.1 (0–0.7)	0.08 (0–0.8)	0.04 (0–7.2)	0.2 (0–0.8)	0.2 (0–0.9)	0.027	0.094	0.027
*Fusobacteriota*	0.0008 (0–4.2)	0 (0–1.2)	0.004 (0–0.4)	0 (0–0.3)	0 (0–0.3)	0 (0–0.2)	0.421	0.634	0.421
*Candidatus Patescibacteria*	0 (0–3.2)	0 (0–0.01)	0 (0–0)	0 (0–0.2)	0 (0–0.1)	0.01 (0–0.2)	0.411	0.364	0.411
*Synergistetes*	0 (0–0.05)	0 (0–0.3)	0 (0–0.2)	0 (0–0.04)	0 (0–0)	0 (0–0)	0.912	1	0.912
*Cyanobacteriota*	0 (0–0.1)	0 (0–0.4)	0 (0–0.09)	0 (0–0.03)	0 (0–0.2)	0 (0–0.2)	0.523	0.334	0.523
Diversity									
Fisher index	4.9 (2.9–8.3) ^a^	5.4 (2.7–11.9) ^b^	4.8 (2.81–8.6) ^a^	6.5 (3.0–12.9)	6.8 (4.3–13.5)	6.2 (2.9–15.0)	<0.001	<0.001	<0.001
Shannon	0.9 (0.09–2.0) ^a^	1.2 (0.1–2.1) ^b^	0.8 (0.3–1.5) ^a^	1.65 (0.3–3.0)	1.85 (1.0–3.0)	1.7 (0.7–2.6)	<0.001	<0.001	<0.001
Inverse Simpson	1.9 (1.0–6.6) ^ab^	2.4 (1.0–5.4) ^a^	1.6 (1.1–2.8) ^b^	2.72 (1.1–12.8)	3.36 (1.6–11.5)	3.1 (1.3–7.8)	<0.001	<0.001	<0.001
Pielou’s evenness	0.25 (0.03–0.6) ^a^	0.32 (0.04–0.6) ^b^	0.22 (0.08–0.4) ^a^	0.43 (0.09–0.7)	0.48 (0.3–0.7)	0.44 (0.2–0.6)	<0.001	<0.001	<0.001
Species richness	37.2 (24–59) ^a^	40.7 (22–80) ^b^	36.4 (23–61) ^a^	48 (25–86)	49.5 (33–89)	46 (24–98)	<0.001	<0.001	<0.001
Simpson	0.37 (0.02–0.9) ^ab^	0.48 (0.03–0.8) ^a^	0.32 (0.08–0.6) ^b^	0.63 (0.09–0.9)	0.7 (0.4–0.9)	0.68 (0.2–0.9)	<0.001	<0.001	<0.001

Data are expressed as median and range. The *p*-value was calculated using the independent sample median test. Different letters mean significant differences (*p* < 0.05) and were calculated with an independent sample median test adjusted by the Bonferroni correction for multiple tests.

**Table 2:** The distribution of genera in the INNOVA 2020 study at 6 and 12 months of infant’s age.

Genus	6 Months			12 Months			*p*-Values		
	BF (n = 52)	STD (n = 55)	INN (n = 52)	BF (n = 59)	STD (n = 63)	INN (n = 60)	BF	STD	INN
*Bifidobacterium*	78.7 (3.6–98.9) ^a^	68.5 (0.8–98.3) ^b^	82.7 (4.8–95.7) ^a^	53.8 (4.0–95.2)	48.0 (5.4–77.2)	51.2 (15.5–89.0)	<0.001	0.006	<0.001
*Clostridium sensu stricto* 1	0.5 (0–72.4) ^a^	0.5 (0–88.5) ^a^	1.4 (0.01–16.9) ^b^	0.6 (0.01–26.0)	0.8 (0–27.7)	1.2 (0.02–34.3)	0.653	0.156	0.850
*Collinsella*	0.4 (0.03–50.9)	0.3 (0.01–54.9)	0.6 (0–63.7)	2.6 (0.02–44.7)	1.9 (0.02–34.1)	5.9 (0.04–33.4)	0.009	0.156	0.014
*Blautia*	0.06 (0–12.3)	0.1 (0–41.4)	0.05 (0–13.3)	0.4 (0–7.7)	0.8 (0–24.5)	0.5 (0–9.5)	<0.001	0.001	<0.001
*Ruminococcus* *gnavus* group	0.2 (0.01–41.0) ^a^	1.6 (0–46.7) ^b^	0.2 (0–73.9) ^a^	0.5 (0.02–15.9)	0.8 (0.06–18.4)	0.8 (0.04–14.0)	0.113	0.400	0.037
*Clostridioides*	0.04 (0–5.8) ^a^	0.3 (0–24.3) ^b^	0.03 (0–39.7) ^a^	0.02 (0–2.1)	0.02 (0–0.5)	0.04 (0–1.6)	0.772	0.001	0.850
*Akkermansia*	0.06 (0–10.9)	0.05 (0–66.1)	0.04 (0–35.6)	0.04 (0–14.6)	0.05 (0–23.3)	0.06 (0–30.5)	0.460	0.967	0.343
*Eggerthella*	0.2 (0–5.2)	0.5 (0–15.5)	0.09 (0–5.7)	0.3 (0–7.2)	0.3 (0–3.2)	0.2 (0–2.5)	0.692	0.642	0.126
*Flavonifractor*	0.04 (0–13.4) ^a^	0.2 (0–9.6) ^b^	0.03 (0–3.2) ^a^	0.1 (0–1.8)	0.2 (0–1.2)	0.1 (0–2.2)	0.194	0.911	0.001
*Subdoligranulum*	0.02 (0–19.5)	0.02 (0–13.6)	0 (0–0.2)	0.05 (0–7.3)	0.07 (0–24.7)	0.1 (0–24.8)	0.048	0.010	<0.001
*Rothia*	0.04 (0–1.2) ^a^	0.02 (0–0.3) ^a^	0.06 (0–0.6) ^b^	0.03 (0–0.5)	0.005 (0–1.6)	0.02 (0–0.5)	0.607	0.175	0.009
*Faecalibacterium*	0.04 (0–5.5)	0.07 (0–27.1)	0.05 (0–3.6)	5.0 (0–18.7)	3.2 (0–22.0)	1.5 (0.02–36.5)	<0.001	<0.001	<0.001
*Lachnoclostridium*	0 (0–17.3) ^a^	0.06 (0–4.1) ^b^	0 (0–3.5) ^a^	0.1 (0–12.8)	0.3 (0–6.0)	0.2 (0–2.6)	<0.001	<0.001	<0.001
*UBA1819*	0 (0–12.5)	0 (0–16.6)	0 (0–2.5)	0.02 (0–1.4)	0.4 (0–3.2)	0.02 (0–3.4)	0.101	0.134	0.126
*Anaerostipes*	0.06 (0–6.2)	0.08 (0–6.6)	0.03 (0–3.0)	1.0 (0–17.7)	1.4 (0.04–9.8)	0.6 (0–9.5)	<0.001	<0.001	<0.001
*Escherichia-Shigella*	0.2 (0–0.9) ^a^	0.1 (0–0.7) ^a^	0.3 (0–1.0) ^b^	0.02 (0–0.5)	0 (0–0.3)	0.02 (0–0.4)	<0.001	<0.001	<0.001
*Streptococcus*	0.09 (0–1.4) ^a^	0.01 (0–0.9) ^b^	0.05 (0–0.7) ^a^	0.05 (0–0.6)	0.04 (0–0.8)	0.05 (0–0.5)	0.113	0.037	0.850
*Ruminococcus*	0 (0–0.2)	0 (0–0.7)	0 (0–0.2)	0.02 (0–10.3)	0.01 (0–6.9)	0.04 (0–5.5)	0.047	<0.001	0.001
*Eubacterium hallii* group	0 (0–9.7)	0.01 (0–6.0)	0.01 (0–1.2)	0.2 (0–11.7)	0.06 (0–19.1)	0.08 (0–6.5)	0.008	<0.001	0.001
*Ruminococcus torques* group	0.01 (0–1.1)	0.03 (0–19.7)	0.01 (0–9.9)	0.2 (0–19.9)	0.3 (0–9.8)	0.1 (0–8.9)	<0.001	<0.001	<0.001
*Veillonella*	0.1 (0–0.7)	0.1 (0–0.8)	0.2 (0–0.8)	0.02 (0–0.7) ^a^	0.01 (0–0.7) ^a^	0.09 (0–0.5) ^b^	0.018	<0.001	0.014
*Roseburia*	0 (0–0.07)	0 (0–0.09)	0 (0–0.08)	0(0–2.0)	0.01 (0–2.4)	0.005 (0–1.4)	<0.001	<0.001	<0.001
*Bacteroides*	0.01 (0–0.9)	0.07 (0–0.6)	0.1 (0–0.9)	0.2 (0–0.6)	0.2 (0–0.6)	0.2 (0–0.5)	0.018	0.072	0.570
*Enterococcus*	0.01 (0–0.4)	0.03 (0–1.0)	0.06 (0–0.8)	0(0–0.5)	0 (0–0.4)	0 (0–0.4)	0.045	<0.001	<0.001
*Eubacterium*	0 (0–5.9)	0 (0–2.5)	0 (0–1.6)	0(0–0.2)	0 (0–1.0)	0 (0–1.2)	0.387	0.115	1

Data are expressed as median and range. The *p*-value was calculated using the independent sample median test. Different letters mean significant differences (*p* < 0.05) and were calculated with an independent sample median test adjusted by the Bonferroni correction for multiple tests.

## Data Availability

A reasonable request should be made to the corresponding author for access to the datasets used and/or analyzed in the current study.

## Supplementary Materials

The following supporting information can be downloaded at https://www.mdpi.com/article/10.3390/ijms24087392/s1.
