# Supplementary material for: Innova 2020: A Follow-Up Study of the Fecal Microbiota of Infants Using a Novel Infant Formula between 6 Months and 12 Months of Age
Source: Int J Mol Sci. 2023 Apr 17;24(8):7392. doi: 10.3390/ijms24087392 (PMC10139017; doi:10.3390/ijms24087392)
Supplement: Supplementary file 1 [file ijms-24-07392-s001.zip › ijms-2286698-supplementary.pdf]

Innova 2020: A Follow-Up Study of the Fecal Microbiota of Infants Using a Novel Infant Formula between 6 Months and 12 Months of Age

Julio Plaza-Diaz [1,2,3,*,†], Francisco Javier Ruiz-Ojeda [1,2,4,5,†], Javier Morales [6], Rafael Martín-Masot [5,7], Eric Climent [8], Ángela Silva [8], Juan F. Martinez-Blanch [8], María Enrique [8], Marta Tortajada [8], Daniel Ramon [8], Beatriz Alvarez [8], Empar Chenoll [8] and Ángel Gil [1,2,4,9,*]

[1] Department of Biochemistry and Molecular Biology II, School of Pharmacy, University of Granada, 18071 Granada, Spain
[2] Instituto de Investigación Biosanitaria IBS.GRANADA, Complejo Hospitalario Universitario de Granada, 18014 Granada, Spain
[3] Children's Hospital of Eastern Ontario Research Institute, Ottawa, ON K1H 8L1, Canada
[4] RG Adipocytes and Metabolism, Institute for Diabetes and Obesity, Helmholtz Diabetes Center at Helmholtz Center Munich, Neuherberg, 85764 Munich, Germany
[5] Institute of Nutrition and Food Technology "José Mataix", Centre of Biomedical Research, University of Granada, Avda. del Conocimiento s/n. Armilla, 18016 Granada, Spain
[6] Product Development Department, Alter Farmacia SA, 28880 Madrid, Spain
[7] Pediatric Gastroenterology and Nutrition Unit, Hospital Regional Universitario de Málaga, 29010 Málaga, Spain
[8] ADM-BIOPOLIS, Scientific Park Universitat de València, 46980 València, Spain
[9] CIBEROBN (CIBER Physiopathology of Obesity and Nutrition), Instituto de Salud Carlos III, 28029 Madrid, Spain
* Correspondence: jrplaza@ugr.es (J.P.-D.); agil@ugr.es (Á.G.); Tel.: +34-695466922 (A.G.)
† These authors contributed equally to this work.

Supplementary Table S1. Nutritional composition of the standard infant formula (STD) and study formula (INN).

Composition	STD formula			INN formula		
	100 g	100 mL	100 kcal	100 g	100 mL	100 kcal
Energy	514	67		489	67	
Energy	2152	280		2046	279	
Total Fat	27	3.5	5.2	25.6	3.5	5.2
Linoleic acid (ω-6)	3.79	493	736	3.147	427	641
α-linolenic acid (ω-3)	500	65	97	387	53	79
Araquidonic acid AA (ω-6)	53	6.9	10	118	16	24
Docosahexaenoic acid DHA (ω-3)	53	6.9	10	118	16	24
Carbohydrates	56.4	7.3	11	54.6	7.4	11.1
Total sugars	56.4	7.3	11	53	7.2	10.8
Lactose	55	7.2	10.7	53	7.2	10.8
Fiber	1.3	0.18	0.22	1.7	0.22	0.33
Proteins	10.6	1.4	2.1	9.4	1.3	1.9
Whey proteins	6.4	0.83	1.2	6.6	0.9	1.3
Caseins	4.2	0.55	0.82	2.8	0.38	0.57
α-casein	20 % of total protein			8% of total protein		
β-casein	14 % of total protein			18 % of total protein		

Supplementary Figure S1. Correlations between bacterial diversity indices, bacterial variables, SCFAs levels, metabolic traits and clinical outcomes A. Breastfed group, B. Standard formula group, C. Innova formula group

A

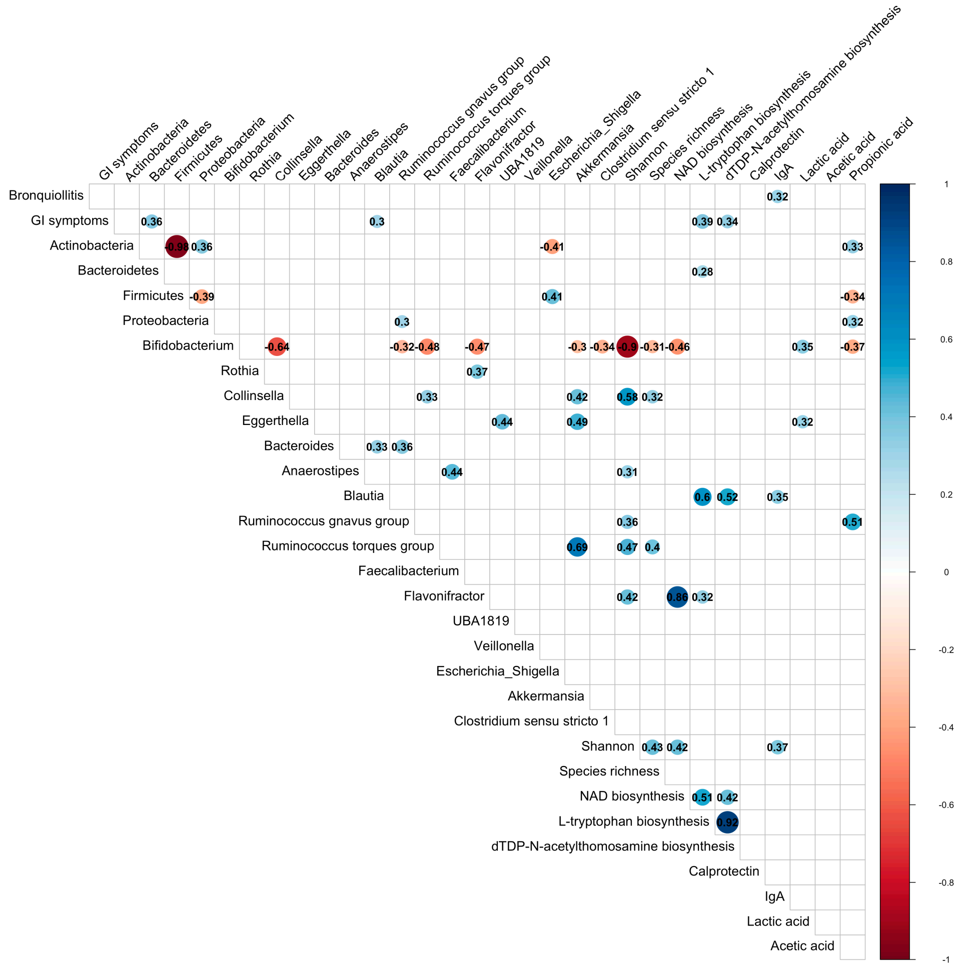

B

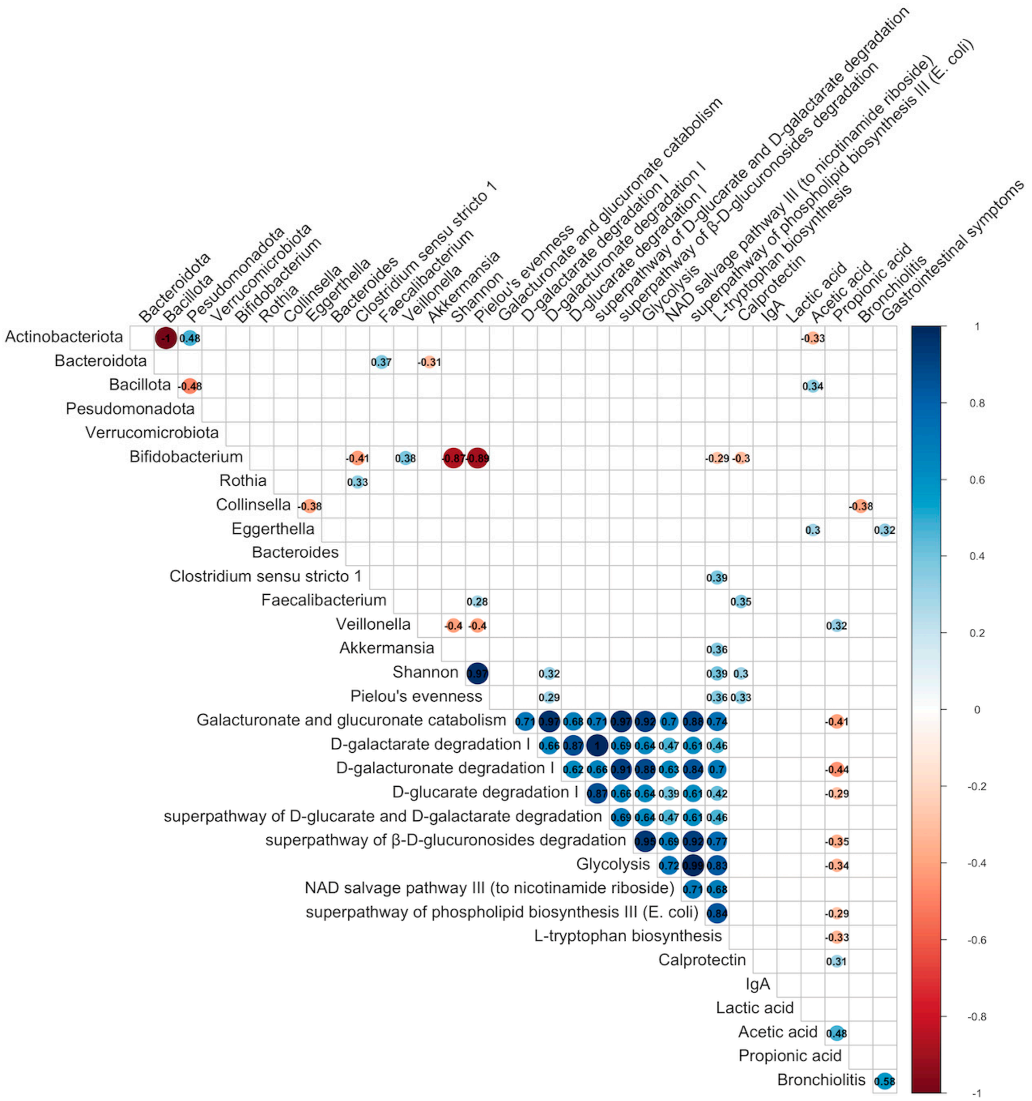
Bacteroidota
Bacillota
Pesudomonadota
Verrucomicrobiota
Bifidobacterium
Rothia
Collinsella
Eggerthella
Bacteroides
Clostridium sensu stricto 1
Faecalibacterium
Veillonella
Akkermansia
Shannon
Pielou's evenness
Galacturonate and glucuronate catabolism
D-galactarate degradation I
D-galacturonate degradation I
D-glucarate degradation I
superpathway of D-glucarate and D-galactarate degradation
superpathway of β-D-glucuronosides degradation
Glycolysis
NAD salvage pathway III (to nicotinamide riboside)
superpathway of phospholipid biosynthesis III (E. coli)
L-tryptophan biosynthesis
Calprotectin
IgA
Lactic acid
Acetic acid
Propionic acid
Bronchiolitis
Gastrointestinal symptoms
Actinobacteriota
1
0.8
0.6
0.4
0.2
0
-0.2
-0.4
-0.6
-0.8
-1

C

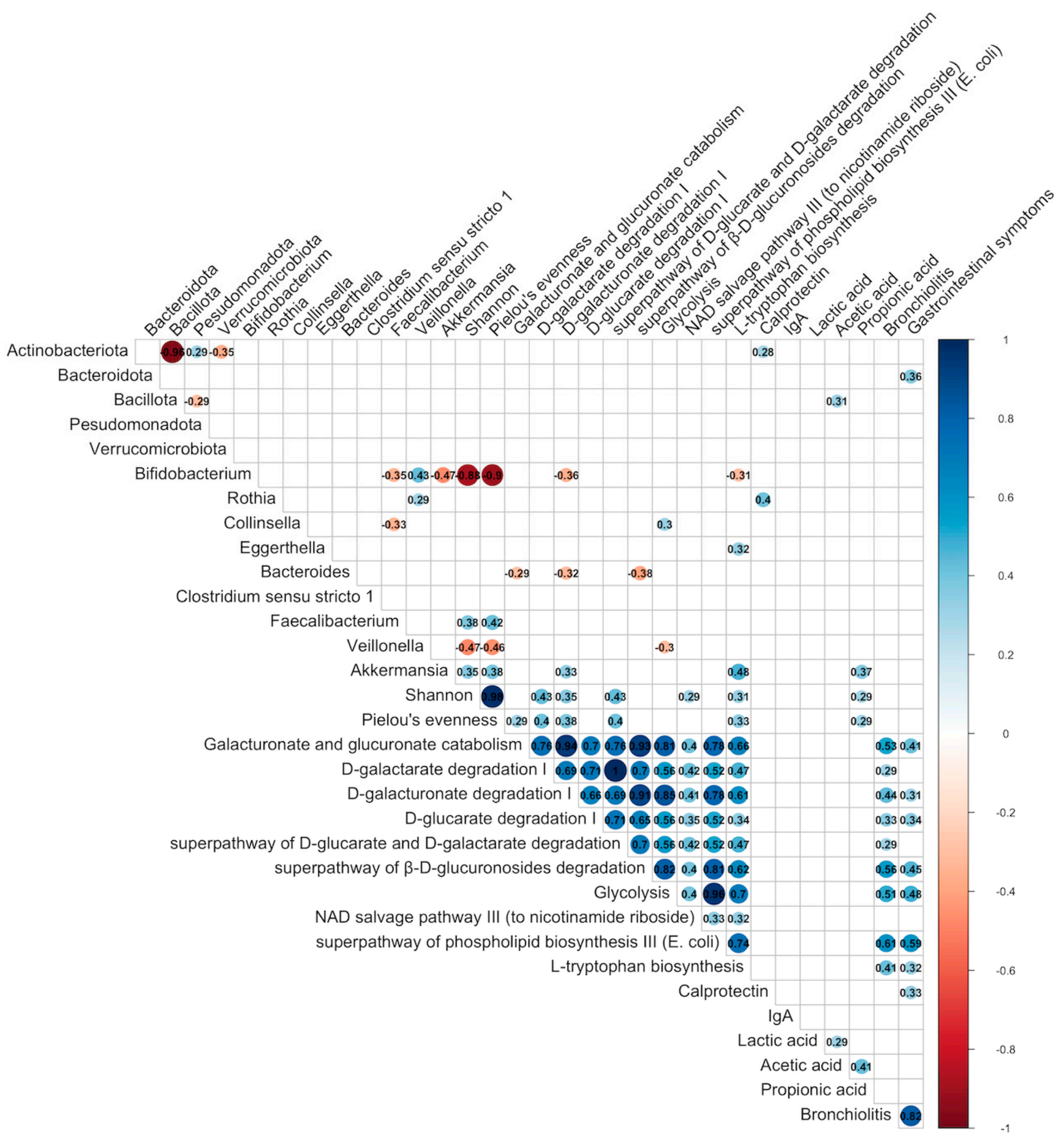
Actinobacteriota
Bacteroidota
Bacillota
Pesudomonadota
Verrucomicrobiota
Bifidobacterium
Rothia
Collinsella
Eggerthella
Bacteroides
Clostridium sensu stricto 1
Faecalibacterium
Veillonella
Akkermansia
Shannon
Pielou's evenness
Galacturonate and glucuronate catabolism
D-galactarate degradation I
D-galacturonate degradation I
D-glucarate degradation I
superpathway of D-glucarate and D-galactarate degradation
superpathway of β-D-glucuronosides degradation
Glycolysis
NAD salvage pathway III (to nicotinamide riboside)
superpathway of phospholipid biosynthesis III (E. coli)
L-tryptophan biosynthesis
Calprotectin
IgA
Lactic acid
Acetic acid
Propionic acid
Bronchiolitis
Gastrointestinal symptoms
